# Coinage Metals Doped ZnO Obtained by Sol-Gel Method—A Brief Review

**DOI:** 10.3390/gels9050424

**Published:** 2023-05-18

**Authors:** Cristina Maria Vlăduț, Oana-Cătălina Mocioiu, Elena Mirabela Soare

**Affiliations:** Institute of Physical Chemistry Ilie Murgulescu of the Romanian Academy, 202 Splaiul Independenţei, 060021 Bucharest, Romania

**Keywords:** ZnO, coinage metals, sol-gel method, gold, silver, copper

## Abstract

ZnO is one of the most studied oxides due to its nontoxic nature and remarkable properties. It has antibacterial and UV-protection properties, high thermal conductivity, and high refractive index. Various ways have been used to synthesize and fabricate coinage metals doped ZnO, but the sol-gel technique has received a lot of interest because of its safety, low cost, and facile deposition equipment. Coinage metals are represented by the three nonradioactive elements of group 11 of the periodic table: gold, silver, and copper. This paper, which was motivated by the lack of reviews on the topic, provides a summary of the synthesis of Cu, Ag, and Au-doped ZnO nanostructures with an emphasis on the sol-gel process and identifies the numerous factors that affect the morphological, structural, optical, electrical, and magnetic properties of the produced materials. This is accomplished by tabulating and discussing a summary of a number of parameters and applications that were published in the existing literature over the previous five years (2017–2022). The main applications being pursued involve biomaterials, photocatalysts, energy storage materials, and microelectronics. This review ought to serve as a helpful reference point for researchers looking into the many physicochemical characteristics of coinage metals doped ZnO, as well as how these characteristics vary according to the conditions under which experiments are conducted.

## 1. Introduction

Zinc oxide is semiconductor oxide, which has distinct optical, electrical, and mechanical properties. ZnO is a multifunctional material that has many applications in industries such as medicine, consumer products, electronics, environmental remediation, and optical and electrical devices because of its special features [1,2,3,4,5]. There are three categories of synthesis techniques: physical, chemical, and biological (green route). Pulsed laser deposition, magnetron sputtering, electrodeposition, and electron beam evaporation are examples of physical approaches [6,7,8,9,10]. The hydrothermal, solvothermal, sol-gel, chemical bath deposition, wet chemical process, spray pyrolysis, microemulsion, and precipitation methods are included in the chemical synthesis approach [11,12,13,14,15,16,17,18,19,20]. Plant or microbial extracts are biocompatible and can act as capping agents to stabilize the NPs in the green or biogenic synthesis of metal oxide nanomaterials [21,22].

According to the literature, several methodologies are being created to enable these nanostructures to have the desired properties. As a result, choosing an effective synthesis method is crucial, and this is an essential factor that greatly affects the effectiveness of the generated nanocrystalline materials.

According to Dimitriev et al. [23], the chemical process known as sol-gel was once used to create glass and ceramic materials. Sol-gel is now mainly utilized to create metal oxide nanoparticles. The process consists of both aqueous and non-aqueous steps that use water and organic solvents. By virtue of the water molecules, aqueous sol-gel chemistry transforms the precursor (often an inorganic metal salt or metal alkoxide) into an inorganic solid.

**Figure 1:**
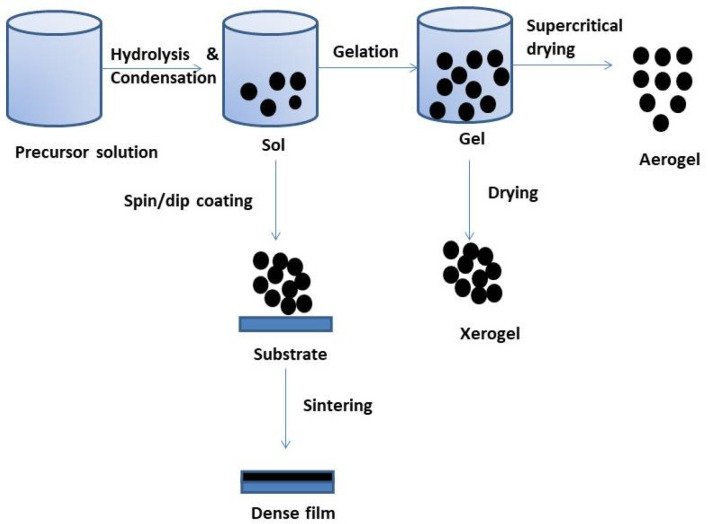
Sol-gel process.

Non-aqueous sol-gel chemistry, on the other hand, thermally decomposes the precursor, which is typically a metal acetylacetonate or acetate, an organometallic compound, a metal alkoxide, or an inorganic metal salt. Sol-gel was chosen because of its extensive use of low temperatures, which was more cost-effective than physical approaches. Moreover, this method provides the appropriate rate of thermal stability, high flexibility of reproducible crystal formation, improved control of particle size and shape to suit a wide variety of applications, and fairly low setup costs for the apparatus [24,25]. A chemical solution (sol) or colloidal particles are used in sol-gel to generate an interconnected network (gel) using an aqueous or organic solvent. As shown in Figure 1, the procedure entails a number of processes, including hydrolysis and polycondensation, gelation, aging, drying, and crystallization.

The structure of the generated gel is influenced by the chosen drying method. Moreover, the drying process and relative humidity have an impact on the type of nanoparticles that will form. For example, low humidity may be necessary for nano-films to generate stable samples. As previously mentioned, the calcination of the sample also affects the morphology of the sample generated [26,27].

Within the vast transition-metal family, group 11 coinage metals (Cu, Ag, and Au) have attracted a great deal of attention and are the subject of substantial research. Fundamental research and technological advancement depend heavily on the design and synthesis of these coinage metals. Introducing new nanostructures and enhancing existing ones for enhanced functionality are the ultimate goals of these manufacturing techniques. Improving the characteristics of metal nanostructures and hence their prospective applications mostly depends on nanoscale manipulation of shape, size, composition, architecture, and structure.

Coinage metal nanostructures have notable qualities, such as catalytic, electrical, optical, and chemical properties that depend on size and form. Each of these characteristics has stimulated intensive research into the development and production of coinage metal nanostructures as well as the possible uses for these structures. Doping ZnO with Cu, Ag, and Au differs from doping with other transition metals because they have a higher tendency to occupy both interstitial and substitutional sites within the lattice. In contrast, other transition metals typically occupy only interstitial sites, leading to a lower concentration of dopants within the lattice. This unique doping behavior of Cu, Ag, and Au in ZnO can result in significant changes in the optical, electrical, and structural properties of the material.

This review intends to present a current summary of the progress made in coinage metals doped ZnO nanostructures using a straightforward and affordable technology, such as the sol-gel method, as described in literature research from 2017 to 2022. The preparation method and the morphological characteristics of the doped nanostructures, as well as their properties, such as electrical, optical, and photocatalytic properties, are reviewed and discussed in this paper.

## 2. Synthesis of Coinage Metals Doped ZnO

The raw material preparation and growth processes play a crucial role in determining the material properties. Due to the various production methods, the same material has various physicochemical properties. The experimental setup has a significant impact on their properties, and the solvents, additives, substrate, annealing, and coating processes are only a few of the variables that affect how well this process works [28,29,30,31,32,33,34,35,36,37,38,39,40,41].

The factors that affect the sol-gel process used to create coinage metals doped ZnO, such as the type of solvent used, the additive species used, the source of the precursor and its concentration, the stirring and aging time of the sol and the type of substrate, are all summarized in Table 1 [42,43,44,45,46,47,48,49,50,51,52,53,54,55,56,57,58,59,60,61,62,63]. 

The sol is created by either hydrolysis or polymerization processes once the reagents and precursors are combined, and after being heated, the sol is changed into a gel. The gel could be dried and turned into a powder, or it could be coated on the substrates and calcined to create thin films in a variety of shapes, including nanorods, nanotubes, nanospheres, nanoflakes, nanofibers, and nanoribbons. The numerous precursors, solvents, and additives utilized in the sol-gel process for creating the coinage metals doped ZnO nanostructures are listed in the following section.

### 2.1. Sol-Gel Sample Preparation

The experimental setup for the sol-gel method is very simple. Sol-gel’s main components are classified as follows:

#### 2.1.1. Precursors

By altering the precursor’s concentration, the sol-gel properties can be changed. Zinc acetate dehydrate is the zinc precursor that is most frequently used (ZAD; Zn(CH_3_COO)_2_ 2H_2_O) [42,43,44,45,46,47,48,49,50,51,52,53,55,56,57,58,59,60,61,62,63], while other sources, such as zinc chloride [54], are less common. As copper precursors, several sources are utilized: copper acetate (CA; Cu (CH_3_COO)_2_) [43,45,48,49], copper acetate monohydrate (CAM; Cu (CH_3_COO)_2_ H_2_O) [42,44,47] and copper acetate tetrahydrate (CAT;Cu(CH_3_COO)_2_ 4H_2_O) [46]. The most commonly used silver dopant is silver nitrate [50,51,53,54,55,56,57,58], followed by silver acetate [52]. While as gold precursors the following sources are used: gold nitrate [59], HAuCl_4_ ∙3H_2_O [60], HAuCl_4_ 4H_2_O [61], gold(III) chloride hydrate HAuCl_4_∙H_2_O [62], evaporated gold wires [63]. 

#### 2.1.2. Additives

The concentration of additives affects the structural characteristics, particle size, and growth mode of the nanostructures [64]. To speed up the complete dissolution of the precursors in the solvents and the creation of a stable sol, additives are used [64]. The most commonly used additives are monoethanolamine [47,49,56,61,62,63] and triethanolamine (TEA) [57,60].

#### 2.1.3. Solvents

Making sure that the precursors are soluble in the solvent medium and easily break down into volatile compounds is crucial for the preparation of nanostructures [65]. There are various types of solvents used: ethanol (EtOH; C_2_H_5_OH) [47,48,52,56,60,61,62], methanol (MeOH; CH_3_OH) [42,45,46,55], 2-methoxyethanol [2-ME; CH_3_ O(CH_2_)_2_OH] [49,63] and deionized or distilled water [43,50,57,58,59].

### 2.2. Nanostructures

Nanotechnology creates a wide range of materials at the nanoscale level. Particulate compounds with at least one dimension smaller than 100 nm are included in the broad class of materials known as nanoparticles (NPs) [66]. The general shape of these materials determines whether they are 1D, 2D, or 3D [67]. Semiconductor NPs have broad bandgaps, which leads to a considerable change in their characteristics when the bandgap is tuned. Its distinctive size, shape, and structure also affect its reactivity, toughness, and other properties. These characteristics make them good candidates for a variety of commercial and household applications, including use in catalysts, photonics, electronics, medical devices, energy-based research, and environmental applications [68].

#### 2.2.1. Xerogels

Xerogels are a particular category of solid-formed gel made by slowly drying at room temperature with uncontrolled shrinkage [69]. The characteristics of xerogels typically include increased porosity, a bigger surface area, and extremely small pore diameters. These are made using the sol-gel technique. Different metal alkoxide precursors, water, or ethyl alcohol are needed when making xerogels using the sol-gel process. The preparation of solid oxide networking (also known as the sol-gel transition process) is a direct result of the liquid alkoxide’s hydrolysis and condensation processes [70]. ZnO xerogels produced by the sol-gel transition method are nontoxic materials.

#### 2.2.2. Aerogels Dried in Supercritical Conditions of Ethanol

According to Garcia-Gonzalez et al. [71], aerogels are typically sol-gel materials in which the liquid component of the gel systems has been replaced with the gas to eliminate the integral solid nanostructures free of the collapsing of pores. By volume, these materials contain between 90% and 99% air. The remarkable changes in the gel’s properties are stimulated by the aerogels’ density, which ranges from 1 to 1000 kg/m^3^. Aerogels exhibit multipurpose and distinctive characteristics such as highly specific surface area, dielectric constant, refractive index, sonic velocity, ultrawide adjustable density, and ultralow thermal conductivity, among others, because of their higher porosity and dual structural features of both macroscopic (i.e., condensed state matter) and microscopic scales (i.e., nano-skeleton) [72].

Aerogels are obtained under supercritical conditions. The use of techniques utilizing a supercritical fluid medium is a novel method for creating nanoscaled multifunctional materials, including the controlled production of powders and nanostructured surfaces [73,74,75].

Slimi et al. [42] showed in their synthesis that the Zn and Cu precursors are dissolved in methanol for 15 min while being constantly stirred magnetically to create the aerogels. After these acetates were completely dissociated, the final solution was put into an autoclave (Parr 4848 Reactor) containing 267 mL of ethanol. The mixture was then dried under supercritical ethanol conditions (Tc = 243 °C and Pc = 63 bars). The solvent was removed once the necessary conditions were met, and the autoclave was then left to naturally cool to room temperature. The aerogels were collected and characterized after cooling without the use of any chemicals or heat [42].

#### 2.2.3. Thin Films (Spin-Coating and Dip-Coating Methods)

**Figure 2:**
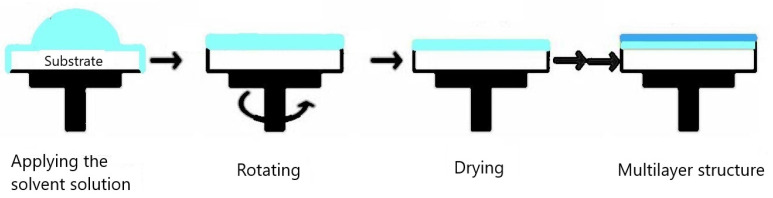
Spin-coating technique.

Due to its low cost and ease of use, ZnO produced using the sol-gel method is frequently used in the spin coating or dip coating processes for the creation of thin films. Even at low temperatures and under controlled circumstances, it can produce uniform nanostructures. The spin coating technique uses drop-casting to apply the sol-gel to the rotating substrate. During rotation, the extra liquid drains from the substrate (Figure 2) and, with a constant speed rotation, enables uniform deposition on the substrate [76]. The deposition is also influenced by additional factors, including coating duration and rotation speed [77]. In the end, the nanoparticles agglomerate through drying after the solvent evaporates, leaving a solid, dry film on the substrate.

Synthesis of coinage metals doped ZnO thin films obtained by the spin-coating technique is presented in previous articles [43,44,49,55,56,61,63].

**Figure 3:**
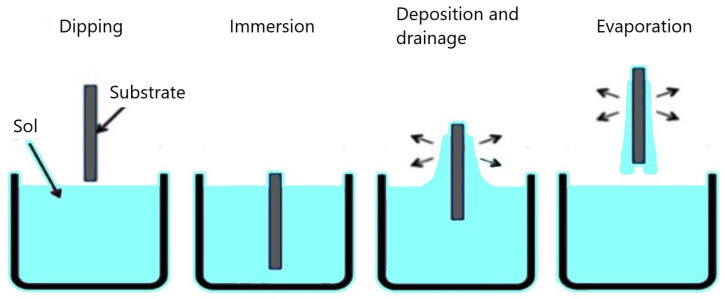
Dip-coating technique.

In the case of the dip-coating technique, a substrate is immersed in the sol-gel suspension and then pulled out under extremely controlled circumstances at a specific speed (Figure 3). The coating sample goes through similar steps of heat treatment as in the spin coating process in order to produce a proper film. Details about the dip-coating technique of the coinage metals doped ZnO thin films can be found in previous articles [46,62].

#### 2.2.4. Substrate

The substrate materials have an impact on the growth mode and morphology of the films [78]. The most popular substrate for coinage metals doped ZnO thin film deposition using the sol-gel method is glass [43,44,46,49,51,55,56,59,62]. The deposition of thin films on a variety of substrates, including indium tin oxide (ITO) [43], silicon [49,63], alumina substrates equipped with interdigitated Au electrodes [50], and inter-digitated electrode (IDE) patterned silicon substrates [61], is also made possible by the spin and dip coating techniques.

#### 2.2.5. Heat Treatment

For the synthesis of thin films, two heat treatment procedures are used: the pre-heat treatment, which removes the solvent and leaves behind a dry solid film, and the annealing procedure, which causes the nanoparticles to coalesce and agglomerate. Pre-heating is dependent on the additives and solvents and needs to be higher than the boiling points of the solvents [78]. For the growth of (002) oriented films during the evaporation and removal of organic compounds, some additive-solvent combinations, such as monoethanolamine-2-methoxyethanol (MEA-2-ME), need higher temperatures (between 250 and 500 °C). In contrast, other solvent combinations, such as diethanolamine-2-propanol (DEA-2-PrOH), only need a low temperature (between 70 and 150 °C). The direction of the crystallites and the uniform crystallization of the films are significantly influenced by the annealing temperature, which typically ranges from 400 to 600 °C [78,79].

## 3. Structure and Morphology

The analytical technique X-ray diffraction (XRD) is largely used to confirm the inclusion of defects into the host lattice and to provide structural and crystallographic details about a material. Prior to other characterizations, it offers essential information about the structural characteristics of the materials.

Most of the information in the literature shows that the incorporation of Cu and Au into ZnO does not change the wurtzite hexagonal structure of ZnO [42,45,48,49,59,62]. 

**Figure 4:**
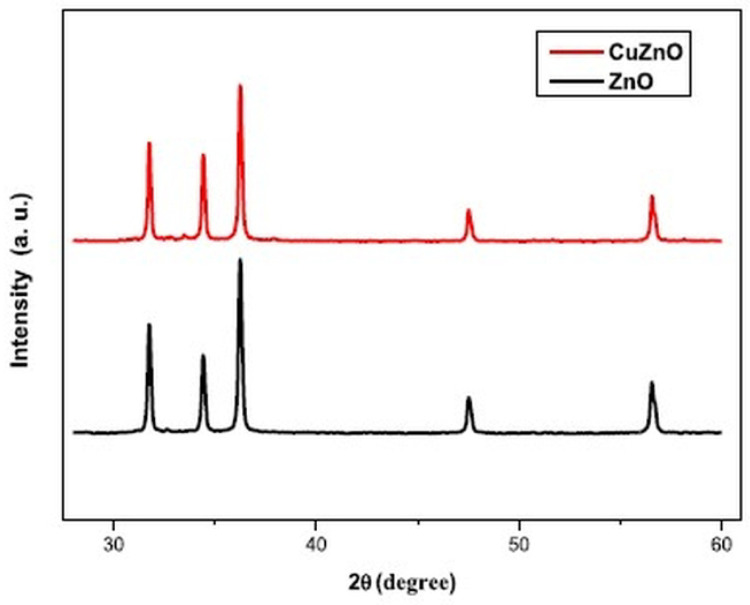
X-ray diffraction peaks of pristine and Cu-doped ZnO nanoparticles (Reprinted with permission from Ref. [48]. 2019, Elsevier from Journal of Alloys and Compounds).

Omri et al. [45] and Mahmoud et al. [48] discussed the structure of Cu-doped ZnO nanoparticles. XRD was used to analyze the crystal structure and phase composition of the nanoparticles. Both studies found that Cu^2+^ ions replaced Zn^2+^ ions in the host lattice of ZnO without changing the crystal structure of the ZnO lattice (Figure 4). Proving that as long as the concentration of dopants remains within the solubility limits, the structure does not change. The peak position’s shift was related to the uniform diffusion and replacement of Zn ions in the hexagonal lattice by copper ions. In these studies, Scherrer’s equation was used to estimate the crystallite size of the nanoparticles. Cu doping was found to increase the crystallite size and the nucleation and growth rate of ZnO. The crystallite size increased from 33–34 nm to 43–44 nm as the doping concentration increased from undoped to 3 at% Cu.

However, Slimi et al. [42] reported the appearance of some peaks in the diffraction patterns indicating that copper crystalline clusters were formed. They explained that, at lower Cu doping concentrations, Cu ions could effectively replace Zn ions. However, when Cu concentration increases over 3%, Cu atoms begin to cluster and are segregated as an impurity phase.

**Figure 5:**
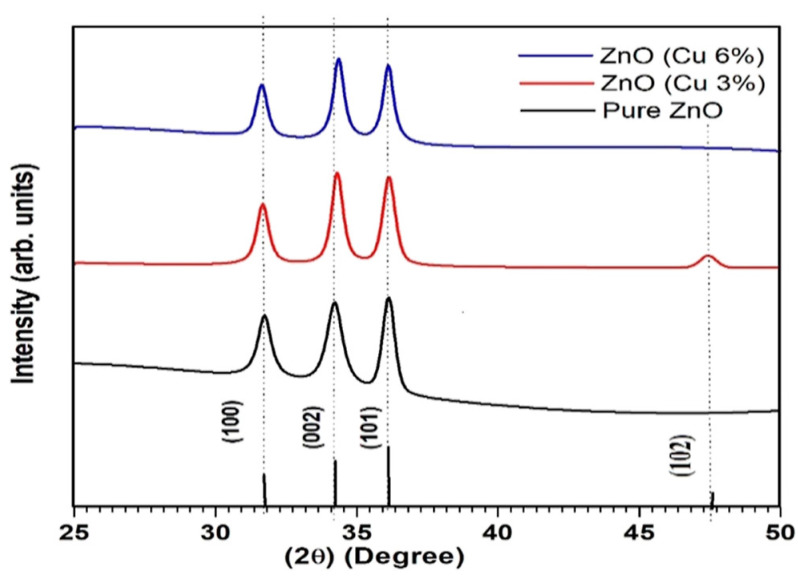
X-ray diffraction peaks of pristine and Cu-doped ZnO films deposited by sol−gel techniques (Reprinted with permission from Ref. [49]. 2022, Creative Commons from ACS Omega).

For Cu-doped ZnO thin films, Joshi et al. [49] stated that all peak positions for pure and doped ZnO samples had wurtzite hexagonal symmetry, and no other Cu-related peaks were observed (Figure 5). They observed that the lattice parameters “a” of 3% Cu-doped ZnO films were slightly smaller than those of undoped ZnO, but for 6% Cu, the parameter was larger, while the interplanar distance “d” decreased with Cu doping. The diffraction peaks were slightly shifted toward higher 2θ with increasing Cu doping. They stated that the valence state of the Cu ions plays an important role, varying the lattice constant along the c-axis (002). The ionic radius of Cu^2+^ (0.073 nm) is slightly less than Zn^2+^ (0.074 nm) ions, while that of Cu^1+^ (0.077 nm) ions is slightly greater than that of Zn^2+^. Thus, the increase in the 2θ diffraction peak indicates that the Cu^2+^ ion has been substituted into the host lattice at the Zn^2+^ position. They observed that with Cu doping, the crystallite size increased, indicating an increase in crystallinity with increasing concentration.

For Ag-doped ZnO samples, the diffraction patterns preserved the wurtzite structure, but the intensity of the peaks was changed upon doping [51,55]. This is assumed to be the disorder effects created by the Ag dopants in the ZnO lattice. Due to that, as the concentration of Ag dopant increased, the crystallinity of the samples decreased.

Vallejo et al. presented in their study [51] the X-ray diffraction pattern of ZnO and Ag-doped ZnO thin films. The diffraction patterns of the doped films retained the characteristic signals of the wurtzite ZnO structure, but there was a noticeable alteration in the intensity of the primary diffraction signal assigned to the (101) plane. This variation in the diffraction intensities was attributed to the incorporation of Ag during the doping process. Furthermore, the changes in the signal intensities were linked to modifications in the grain size resulting from network defects or the presence of oxygen vacancies.

Al-Jawad et al. [55] presented the XRD patterns of Ag-doped zinc oxide thin films with varying levels of Ag doping (0%, 2%, 4%, 6%, and 8%). All films exhibited a hexagonal wurtzite ZnO structure and were polycrystalline. As the concentration of Ag dopant increased, the intensity of all diffraction peaks decreased due to the effects of disorder caused by the Ag ions in the ZnO lattice structure, which affected the kinetics of crystal growth. The decrease in intensity suggested that most of the silver atoms were in the ZnO lattice and caused distortion of the crystalline structure. At 2% Ag doping, a peak with low intensity of orientation (111) due to the silver was observed in addition to the other ZnO peaks. However, for ZnO films doped with 4%, 6%, and 8% Ag, three new peaks corresponding to the (200), (220), and (311) planes of silver appeared. Furthermore, the intensity of (101) and (002) peaks decreased gradually, and their peak positions shifted toward lower values with increasing Ag dopant concentration, indicating a decrease in crystallinity. The crystallite size was calculated and was found to decrease as the Ag dopant level increased. This was due to the substitution of Ag^+^ into the Zn^+^ site.

The structure of Ag-doped ZnO nanoparticles was studied by Sathya et al. [58], and the XRD patterns confirmed the formation of a hexagonal wurtzite structure for all samples. The calcination process positively affected the crystallinity of ZnO nanoparticles, as shown by a very intensive peak in the XRD pattern. Increasing the calcination temperature from 300 to 450 °C gradually decreased crystallite size, which can be attributed to the chemical strain produced by thermo-mechanical forces. Moreover, Ag doping did not significantly affect the position of the diffraction peak, but it strongly impacted the peak intensity. The Ag^+^ ions in the ZnO lattice acted as monovalent dopants and occupied both lattice and interstitial sites due to their higher ionic radius compared to Zn^2+^ ions. Additionally, the dislocation density was found to increase as the crystallite size decreased at 450 °C calcination temperature.

Morphological characteristics provided by atomic force microscopy (AFM), scanning electron microscopy (SEM), field emission scanning electron microscopy (FESEM), and transmission electron microscopy (TEM) offer information about surface roughness and grain size and additional validation to the XRD results. 

According to some authors [42,44,45,48,49,59,62], the grain size of Cu-doped ZnO and Au-doped ZnO increases with the doping concentration, and the samples tend to develop segregation. 

**Figure 6:**
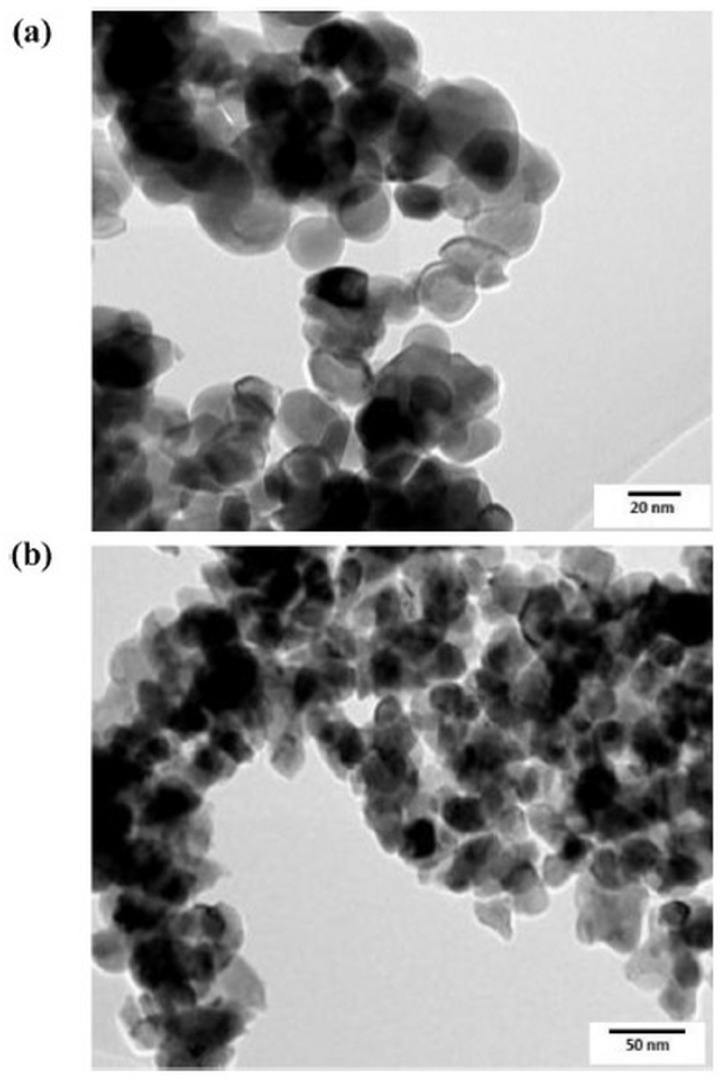
TEM micrograph of (**a**) ZnO and (**b**) Cu-ZO nanoparticles (Reprinted with permission from Ref. [48]. 2019, Elsevier from Journal of Alloys and Compounds).

Omri et al. [45] and Mahmoud et al. [48] discussed the microstructure of Cu-doped ZnO nanoparticles, investigating them with the help of TEM micrographs (Figure 6). The images revealed a spherical morphology and large aggregates for pure ZnO, while Cu doping led to improved segregation and invigorated the growth of ZnO samples. The particle size of the Cu-doped ZnO nanoparticles was larger than that of the pure ZnO nanoparticles in both studies, confirming that higher doping concentrations lead to an increase in grain size.

**Figure 7:**
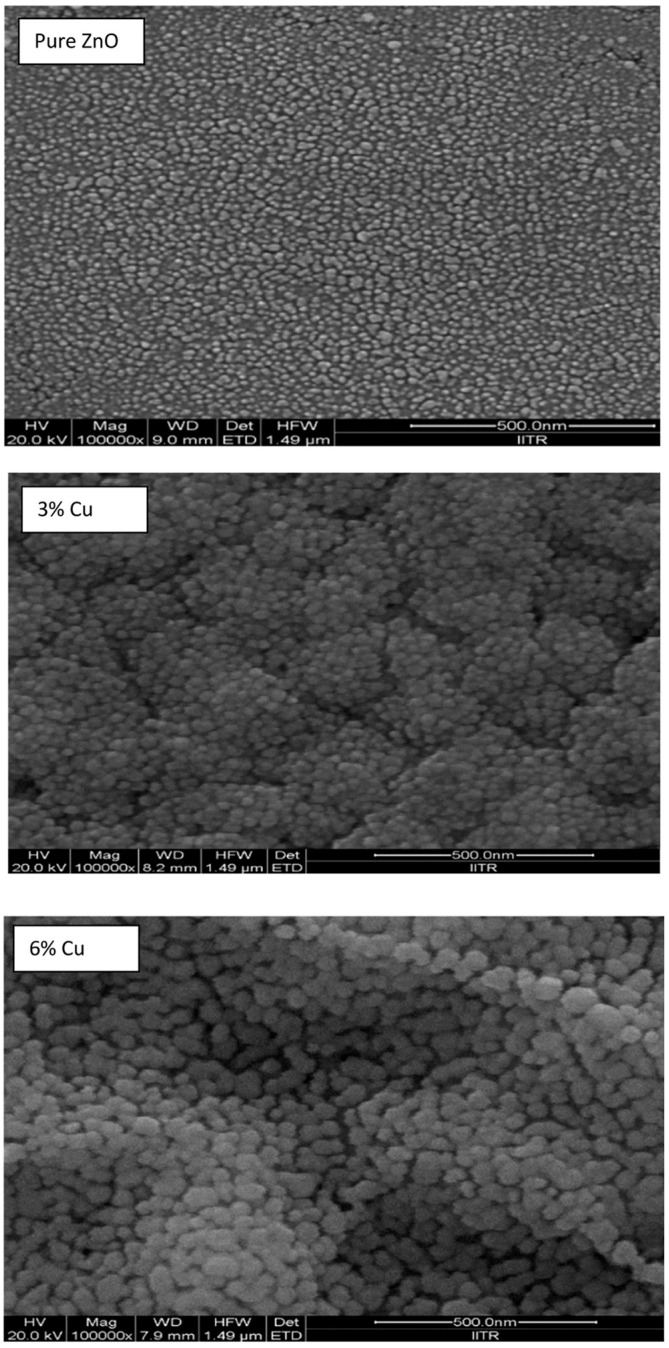
FE-SEM images of pristine and Cu-doped ZnO thin films deposited by the sol−gel method (Reprinted with permission from Ref. [49]. 2022, Creative Commons from ACS Omega).

According to Joshi et al. [49], the crystallite size difference can be due to the different crystal growth rates. The obtained morphology of pure ZnO and Cu-doped ZnO thin films can be observed in Figure 7. The doped nanoparticles had a spherical shape or a combination of spherical and hexagonal shapes. The increased grain size and porosity can be beneficial for gas-sensing applications. Nimbalkar et al. [44] obtained a 3 at% Cu doped ZnO thin film with a porous structure and small nanocrystallites aggregated, which increased the surface area of the thin film for the adsorption of gas.

On the other hand, the incorporation of silver dopants has the reverse effect. Vallejo et al. [51] and Al Jawad et al. [55] reported that the grain size of Ag-doped ZnO films decreased after the doping process. Vallejo et al. [51] showed that both grain size and roughness of the films are smaller for Ag-doped ZnO than for pure ZnO.

The shape and size of the grains depend on the pH, treatment temperature, and the amount of dopant in the samples. In Ag-doped ZnO powders obtained by the sol-gel method, the microstructure consists of uniform grains of 50–100 nm interconnected between them [50,52,53,54,57,58]. Large grains were observed in powders thermally treated at low temperatures (300–350 °C), and smaller grains in powders thermally treated at 450 °C [58].

## 4. Applications

In terms of potential uses, ZnO is one of the most significant metal oxide semiconductors. Comparable to silicon in microelectronics, obtaining ZnO’s nanoscale building blocks and ensuring its outstanding purity is equally essential. Coinage metals doped ZnO can be used as photocatalysts, biomaterials, optical and electrical devices, or as sensors.

### 4.1. Microelectronics

Microelectronics is the study and production of microscopic electrical designs and components. Typically, semiconductor materials are used to construct these devices. Over the last 20 years, outstanding developments in microelectronics have enabled the implementation and realization of digital signal processing (DSP) systems, fast and power-efficient computer systems, biomedical systems, and communication systems. Today’s microelectronic technology includes integrated circuits, thin and thick films, hybrids, and combinations of these [80].

#### 4.1.1. Optical Properties

Optical properties of coinage metals doped ZnO and pure ZnO obtained by an inexpensive sol-gel route synthesis at room temperature were reported. By adding 2% Ag in ZnO nanoparticles, Chitradevi et al. found that the bandgap energy of pure ZnO and Ag-doped ZnO nanoparticles decreases the exciton absorptions from 3.1 eV to 1.6 eV [53]. According to the observed absorption spectra, adding silver dopants causes a noticeable shift to a longer wavelength (a redshift), which is caused by a powerful interaction between zinc oxide and silver. Silver ions may replace zinc ions in ZnO or act as an intermediary atom from the space around the structural unit, as demonstrated by a change in the visible spectrum towards the red domain.

According to Sathya et al. [58], pure ZnO produced using the sol-gel technique has a 3.22 eV bandgap. Upon doping with Ag and raising the temperature from 300 °C to 450 °C, the bandgap reduced from 3.22 eV to 3.15 eV. It is believed that Silver atoms occupy the zinc site in the ZnO lattice, hence lowering the band gap of ZnO. The presence of p-type conductivity in Ag-doped ZnO nanoparticles can also be linked to a decrease in the band gap. The best optical properties were obtained for the Ag-doped ZnO sample thermally treated at 450 °C [58]. The temperature of the thermal treatment of powders obtained by the sol-gel method has a strong influence on the optical properties [58,81,82]. 

Cu-doped ZnO aerogels were studied by Slimi et al. [42]. They reported that the photoluminescence spectra of undoped and Cu-doped ZnO aerogels at room temperature exhibit a broadly visible luminescence (490–650 nm) and a narrow UV emission at 388 nm. Two green emissions (510 and 545 nm) and one orange emission (612 nm) combine to form the visible PL spectrum. Due to Cu impurity-induced non-radiative recombination and the dispersion of excitation radiation by surface-adsorbed dopant atoms, the visible photoluminescence intensity drops as the Cu content rises [42].

According to Nimbalkar et al. [44], as Cu doping concentration increases from 0 to 3 at%, the band gap for thin films decreases from 3.17 to 3.11 eV, exhibiting a small red shift (ΔEg = 0.06 eV). Claiming that the localized d electrons of the metal ion (Zn) that is substituting for the Cu ion are involved in sp-d spin–exchange interactions with the band electrons, which results in this characteristic of the slight red shift in the band gap of metal-doped II-VI semiconductors. The substitution of Cu ions in the ZnO lattice due to exchange interactions is indicated by the band gap’s red shift [44].

Other authors also indicate the decrease of the band gap with increasing the content of the dopant.

Omri et al. [45] reported that the absorbance of Cu-doped ZnO nanoparticles increases with the concentration of Cu doping, revealing that the capacity for absorption increases with Cu atom doping, resulting in an improvement in the efficiency of light exploitation. With the insertion of Cu from 0 to 4 at%, CZO nanoparticles displayed a slight redshift of the absorption band; consequently, the band gap value dropped from 3.34 eV to 3.27 eV [45]. Asikuzun et al. [46] noticed that when the Cu doping level increased, the transmittance values of Cu-doped ZnO films dropped. The improved crystallinity of the Zn1xCuxO films with larger grain sizes was related to the overall reduction in the optical band gap. Cu^2+^ ions at substitution sites enhanced carrier concentration, resulting in a decrease in band-gap energy. The optical band-gap energy ranged from 3.24 to 3.27 eV for the Zn1xCuxO thin films and was roughly 3.28 eV for the undoped ZnO films [46]. According to Xu et al. [47], the samples’ visible absorption is clearly enhanced as a result of the inclusion of Cu. Joshi et al. [49] also noted that as the Cu doping level was increased, the transmittance values of Cu-doped films lowered, and the band gap reduced from 3.14 eV to 3.07 eV. 

Au-doped ZnO-Sm nanoparticle films were studied by Saleem et al. [59]. They demonstrated that the films had an optical transmittance of 25–70% seen between 300 and 800 nm wavelengths. The transmittance for the 1.5 wt% Au-doped ZnO nanoparticle film decreased further to 25% with the rise in Au-doping from 0% to 1.5 wt%. The rough and porous surface of doped films was attributed to the decrease in transmission. As Au content rises, more agglomeration takes place, causing larger particles to be visible. This worsens transparency and increases light absorption. Porosity also rises as a result of an increase in particle size, which enhances dye loading and light absorption. As the absorbance increased due to small and big size nanoparticles in the visible light range, the transmission spectra revealed a declining trend because of the rough and porous surface. These films were employed as photoanodes to improve the performance of DSSCs by limiting the backward flow of electrons (enhancing electron transport) and broadening the spectrum of light absorbed through up- and down-conversion. The cell with 1.5 wt% Au-doped ZnO-Sm photoanode had the best efficiency, coming in at 4.35%, which is around 76% greater than its competitors. According to EIS measurements, the expanded light spectrum and quicker electron transport may be responsible for the improvement in the cell’s performance [59].

According to research by Ouarez et al. [62] on the optical characteristics of ZnO films with 10–30% Au doping, the Au concentration increases ZnO films’ transparency and optical band gap. At room temperature, photoluminescence tests show the presence of four extremely weak emission bands in the visible spectral range and two very strong bands at 373.6 and 385 nm in the UV spectrum. The concentration of Au is observed to boost all visible emissions. Whereas the emission at 385 nm reaches its highest intensity in the film doped with 10 at%, the emission at 373.6 is maximal for the film doped with 20 at% [62].

#### 4.1.2. Electrical Properties

AC measurements are significant tools for examining the dynamic properties of semiconductors and dielectric materials, such as capacitance, conductance, dielectric constant, and dielectric loss tangent. They offer details about a material’s interior in the area of low conductivity [83].

The electrical properties of coinage metals doped ZnO obtained by the sol-gel method have been studied intensively. The dielectric studies for Ag-doped ZnO have shown that the dielectric constant and dielectric loss decrease with frequency and that the temperature and frequency have increased the conductivity [57]. Recent research has linked the type of conduction to the annealing period of various thin films made using the sol-gel spin-coating technique. Xu et al. described the impact of annealing time on Ag-doped ZnO’s electrical behavior in their study [56]. N-type conductivity was seen in thin films that had been annealed for 10 min, while p-type conductivity was visible in films that had been annealed for 20 to 50 min. The authors saw a drop in resistivity for the same composition, followed by a rise in resistivity value [56].

Cu-doped ZnO films were investigated by Liau et al. [43]. As the amount of Cu in the ZnO grew, the results showed that the bandgap energy (Eg) and grain size of the CZO crystals dropped. In the presence of Cu, the CZO crystals’ lattice volume deformed. The CZO sample with a 3 at% Cu addition (3CZO) had the lowest resistivity, according to the analysis. Due to the presence of Cu in ZnO, the levels of the valence band energy (Ev), conduction band energy (Ec), and Fermi energy (EF) were determined and altered. CZO’s reduced resistivity was investigated and linked to energy level changes caused by lattice distortion in CZO crystals [43].

According to several authors [44,45,49], when the Cu doping concentration increases, the conductivity of the films enhances, and the activation energy decreases. In addition, Omri et al. [45] reported that a 3 at% Cu concentration caused the conductivity to reach its highest value.

#### 4.1.3. Sensors

Sensors based on ZnO are widely used because of their high sensitivity and stability, ease of operation and manufacture, short response time, and inexpensive cost. Since they have both chemical and electrical properties, noble metals such as Ag and Au are used as dopants in gas sensors. Ethanol sensors based on Ag-doped ZnO have been obtained, according to Yousefi et al. [50]. The sensor with 3.5 wt% Ag demonstrated the best ethanol selectivity and sensitivity. The catalytic impact of Ag and the development of the Ag-ZnO heterojunction were primarily accountable for the enhancement of the ethanol sensing behavior. Ag-doped ZnO nanoparticles had a particle size that was 30% smaller than pure ZnO nanoparticles, thus improving the contact surface between the particles and the gas and enhancing the sensitivity of the sensor. Due to the size disparity between Ag and Zn, the Ag-doped ZnO crystal lattice exhibits a minor lattice distortion in the XRD patterns, which may offer advantageous adsorption sites for ethanol molecules [50]. Additionally, the sensitivity of Ag-doped ZnO is higher than that of pure ZnO but similar to that of Cu-doped ZnO [84], Au-doped ZnO [85], and Sn-doped ZnO [86].

According to Nimbalkar et al. [44], their gas sensing study showed that the Cu doping concentration and the operating temperature had an impact on the gas response. When the temperature increased, the H_2_S gas sensitivity of CZO thin film sensors grew exponentially. The thin ZnO film with a 3 at% Cu doping exhibits the best response at an operating temperature of 250 °C. For exposure to 50 ppm of H_2_S gas, the CZO sensor has the greatest gas response with 72% of stability. The Cu-doped ZnO thin film can be considered a potential candidate for the detection of H_2_S gas at low concentrations since the CZO sensor demonstrated great selectivity to H_2_S with quick response, recovery time, and good stability (5–50 ppm) [44].

Mahmoud et al. [48] stated that Cu-doped ZnO-based electrodes have outstanding electrochemical characteristics. Moreover, research has shown that Cu doping improves the electrochemical characteristics of ZnO, which has a significant impact on the effectiveness of non-enzymatic glucose sensors. The designed electrode has demonstrated a wide linear range with great sensitivity, reasonable repeatability, a low detection limit, and good interference resistance under ideal conditions. The constructive qualities offered by Cu doping, such as an increased specific surface area that creates huge electro-active sites, good electrical conductivity, and enhanced electro-catalytic activity, may be responsible for the performance enhancement. Finally, a non-enzymatic glucose biosensor was developed and tested to measure glucose in human serum samples with a high degree of accuracy and precision. Cu-ZnO samples are a great candidate for the creation of non-enzymatic glucose sensors due to their easy and affordable method of production and superior properties [48].

Deshwal et al. [61] studied the effect of the sensing behavior of 3% gold (Au) doped ZnO thin films as an acetone sensor. In comparison to pure ZnO thin films, the presented study’s acetone sensors show extremely high sensitivity with quick response and recovery times at a lower ideal working temperature. At an optimal working temperature of 280 °C and a concentration of 500 ppm of acetone vapors, a substantially superior response was seen for the Au-doped ZnO thin films than for the pure ZnO thin films. As a result, the sensing responses are greatly improved, having shorter response and recovery durations when gold is added to ZnO. Therefore, it can be stated that an acetone vapor sensor’s informative parameters are improved by gold doping ZnO thin film [61].

### 4.2. Photocatalyst

The removal of organic pollutants and harmful germs using sunlight has been proposed as a potential application of solar energy utilizing semiconductor-based heterogeneous photocatalysts [87,88,89]. Zinc oxide (ZnO) has received a lot of attention as a significant semiconductor photocatalyst due to its exceptional qualities [90,91,92,93,94]. Furthermore, ZnO has a better photocatalytic performance than TiO_2_ in the photodegradation of organic contaminants [95,96,97,98,99,100,101], despite having a wide band gap (3.37 eV) and high exciton binding energy (60 meV) [102]. The large band gap, however, prevents ZnO from absorbing visible light, which makes up 43% of the solar spectrum, allowing it to solely absorb UV light (around 4% of the solar spectrum) [103]. The efficiency of ZnO as photocatalysts is also reduced by the quick recombination of photogenerated e-hþ pairs. Therefore, ZnO has been altered using various techniques, such as doping with metals and nonmetals and depositing noble metals [104,105,106,107], to increase its visible-light responsiveness and prevent the recombination of e-hþ pairs [108,109].

In addition to changing the reactive sites of ZnO, the deposited noble metal nanoparticles also operate as a cocatalyst for the photodegradation of organic contaminants [110]. By capturing photogenerated electrons and boosting ZnO’s light absorption through surface plasmon resonance (SPR), the noble metal dopants (namely, the plasmonic metals Cu, Ag, and Au) might improve photocatalytic performance. [111]. 

The influence of Ag doping on the photocatalytic properties of ZnO showed an increased interest [51,85]. 

For the 1–5 wt% Ag doped ZnO, Valleho et al. [51] investigated the photocatalytic degradation of methylene blue (MB) under visible irradiation in an aqueous solution. According to the results of the photocatalysis test, doped ZnO thin films had better photocatalytic activity than pure ZnO thin films. This behavior was related to (I) intraband transitions brought on by the insertion of a dopant inside ZnO and (II) grain size reduction. The best photodegradation percentage was observed for 5 wt% Ag-doped ZnO, which was 16.5 times greater than that of pure ZnO.

Under simulated sunshine irradiation on the Cu-doped ZnO-Ag nanoparticles, Xu et al. [47] found that methylene blue (MB) and methyl orange (MO) completely degraded in 40 min. This is accomplished in the following circumstances: low Cu doping level (0.2 wt%) and intermediate Cu concentration (3–5 wt%).

A photocatalyst based on hexagonally shaped Ag-doped ZnO nanoparticles has been created by Jothibas et al. [54]. Methylene blue (MB) degradation photocatalytic efficiencies rose from 60% for pure ZnO to 93% for Ag-doped ZnO nanoparticles [54].

**Figure 8:**
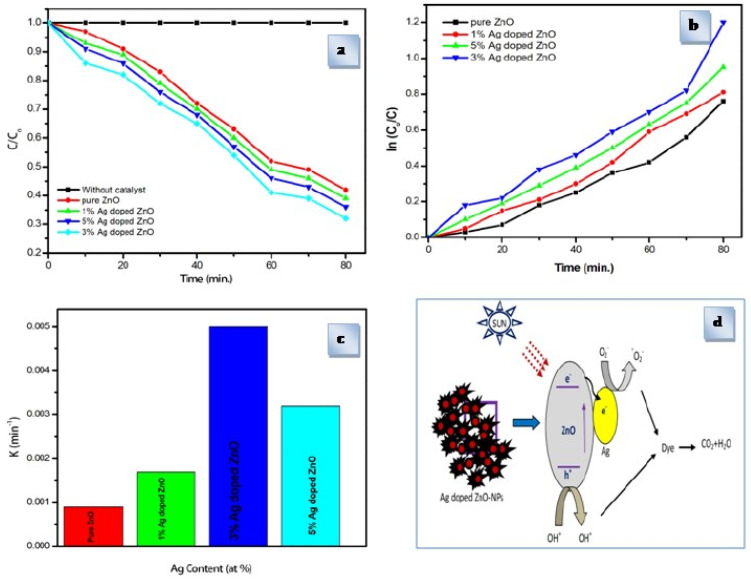
(**a**) Photocatalytic degradation of MB under the irradiation of visible light over the ZnO and Ag-doped ZnO nanoparticles without any catalysts,(**b**) plot of ln(C/C0) as a function of visible light irradiation time for the photocatalysis of MB containing ZnO and Ag-doped ZnO nanoparticles without any catalysts, and (**c**) variation of the observed kinetic constant for MB degradation during the photocatalytic reaction with different Ag contents (**d**) Proposed mechanism for the photocatalytic degradation of MB over the Ag-doped ZnO nanoparticles (Reprinted with permission from Ref. [54]. 2019, AIP Conference Proceedings).

Certain smaller grains have been seen in Ag-doped ZnO nanoparticles, and these can have better photocatalytic activity for MB degradation. Thru this behavior, it was demonstrated that photocatalytic activity and crystal size are directly related. According to the mechanism described by the authors [54], when sunlight falls on ZnO nanoparticles, electrons in the valence band are stimulated to the conduction band, while a similar number of holes are produced in the valence band (Figure 8). The radical species is a powerful oxidant for the mineralization of MB and is produced when the holes react with the MB dye [54].

The Rhodamine B degradation process has been used to assess the photocatalytic activity of Au-doped ZnO nanopowders [60]. Using the sol-gel alcoholic method, pure and Au-doped ZnO powders were created, which were then thermally processed at 400 and 500 degrees Celsius. In the presence of ZnO powder thermally treated at 400 °C, the Rhodamine B conversion was 54% after a 3-h reaction, comparable to TiO_2_. Although the photocatalytic activity of the Au-modified ZnO nanoparticles is higher than that of the pure ZnO nanoparticles, it is still slightly lower than that of the samples heated to 400 °C [60].

### 4.3. Biomaterials

The most important properties of ZnO nanoparticles include biodegradability and low toxicity. ZnO-based nanoparticles can be employed as coatings or as biomaterials in implants since they provide strong antibacterial properties. It is well known that silver (Ag) is a nontoxic and antibacterial material. Due to extensive knowledge in the field, interest in Ag-doped zinc oxide investigations has decreased during the last five years, and fewer articles have been published. AL-Jawad et al. [55] tested the antibacterial activity of Ag-doped zinc oxide and pure ZnO against Escherichia coli and Staphylococcus aureus. The findings indicated that both pure and doped ZnO thin films have an antibacterial inhibitory zone against *E. coli* and *S. aureus*. Compared to Gram-negative bacteria, Gram-positive bacteria appeared to be more resistant to pure and Ag-doped ZnO thin films. When the dopant ratio was raised under UV light, the antibacterial activity of the thin films grew progressively. Under dark conditions, the best concentration of silver in the ZnO structure is 6%, and the antibacterial efficacy at this concentration is 99.32% against *E. coli* and 67.92% against *S. aureus* [55].

## 5. Conclusions

This paper provides a summary of the progress made in Cu, Ag, and Au-doped ZnO nanostructures from the last five years (2017–2022) to help researchers look into the many physicochemical characteristics of coinage metals doped ZnO. Due to extensive knowledge in the field, interest in such doped zinc oxide investigations has decreased during the last five years, and fewer articles have been published.

Coinage metals such as gold, silver, and copper are used as dopants due to their notable qualities. Different morphologies and structures can be obtained for these doped ZnO nanostructures in correlation with the desired properties. 

Most of the information in the literature shows that the incorporation of Cu and Au into ZnO does not change the wurtzite hexagonal structure of ZnO. As for Ag-doped ZnO samples, the diffraction patterns preserved the wurtzite structure, but the intensity of the peaks was changed upon doping. This is assumed to be the disorder effects created by the Ag dopants in the ZnO lattice. Due to that, as the concentration of Ag dopant increased, the crystallinity of the samples decreased.

The morphology of the nanostructures depends on the dopant ion. Most often, grains obtained by the sol-gel method are in spherical form or nanorods. According to the literature, the grain size of Cu-doped ZnO and Au-doped ZnO increases with the doping concentration, and the samples tend to develop segregation. On the other hand, the incorporation of silver dopants has the reverse effect; the grain size of Ag-doped ZnO decreases after the doping process. 

Coinage metals doped ZnO can be used as biomaterials, photocatalysts, and microelectronics. Copper and gold dopants are found to increase the sensing properties of zinc oxide, while silver dopants boost the photocatalytic and antibacterial properties.

## Figures and Tables

**Table 1:** Characteristics of coinage metals doped ZnO obtained by sol-gel method.

Dopant	Conc. Dopant (%) Nominal Value	Precursors	Reaction Parameters	Obtained Material	Properties	Ref.
Cu	1–5 at%	Zinc acetate dihydrate, methanol, copper acetate monohydrate	Drying in supercritical conditions of ethanol	Aerogels	Optical	[42]
Cu	1–5 mol%	Zinc acetate dihydrate, naoh, copper acetate, deionized water	60 °C/1 h, 60 °C/2 h	Thin films (spin coating)	Optical and electrical	[43]
Cu	1–3 at%	Zinc acetate dihydrate, ethanol, m-cresol, copper acetate monohydrate	70 °C/1.5 h, 200 °C/5 min, 600 °C/1 h	Thin films (spin coating)	Optical, electrical, H_2_S gas sensing	[44]
Cu	2–4 at%	Zinc acetate dihydrate, methanol, copper acetate	Drying in supercritical conditions of ethanol	Nanoparticles	Optical, electrical	[45]
Cu	0–5 at%	Zinc acetate dihydrate, methanol, acetylacetone, copper acetate tetrahydrate	60 °C/8 h, 600 °C/30 min	Thin films (dip coating)	Optical	[46]
Cu, Ag	0–5 mol% Cu, 3 mol%Ag	Zinc acetate dihydrate, ethanol, ethanolamine, silver nitrate, copper acetate monohydrate	60 °C/2 h, 48 h aging, 80 °C/10 h, 200 °C/0.5 h, 450 °C/3 h	Nanoparticles	Optical and photocatalytic	[47]
Cu	3 at%	Zinc acetate dihydrate, ethanol, copper acetate	Drying in supercritical conditions of ethanol	Nanoparticles	Electrochemical, glucose sensor	[48]
Cu	3 at%, 6 at%	Zinc acetate dihydrate, 2-methoxy ethanol, ethanolamine, copper acetate anhydrous	2 h stirring, 24 h room temperature aging, annealing at 450 °C/2 h	Thin films (spin coating)	Optical, electrical	[49]
Ag	1.5–5.5 wt%	Zinc acetate dehydrate, silver nitrate, Water, ethylene glycol	70 °C/3 h, 120 °C, 500 °C/2 h	Nanoparticles and films	Ethanol gas sensing	[50]
Ag	1 wt% 3 wt% 5 wt%	Zinc acetate dehydrate, ammonium hydroxide, Silver nitrate	85 °C 100 °C 500 °C, 2 h	Thin Films	Photocatalysis	[51]
Ag	1–10 mol%	Zinc acetate dehydrate, silver acetate, Ethanol, ammonium hydroxide	2 h, 80 °C/24 h, 400 °C/3 h	Nanoparticles	Photocatalysis	[52]
Ag	2 mol%	Zinc acetate dehydrate, silver nitrate, Sodium hydroxide, citric acid	60 °C/1 h, 100 °C, 550 °C/2 h	Nanoparticles	Luminescence	[53]
Ag	1–5 mol%	Zinc chloride, Silver nitrate, Ammonia solution	30 °C/2 h, 100 °C, 400 °C/2 h	Nanoparticles	Photocatalysis	[54]
Ag	2–8 mol%	Zinc acetate dehydrate, silver nitrate, methanol, isopropanol	60 °C/1 h, 150 °C, 500 °C/2 h	Thin Films	Antibacterial properties	[55]
Ag	3 mol%	Zinc acetate dehydrate, silver nitrate, Ethanol, mea	400 °C/ 10–50 min	Thin Films	Electrical	[56]
Ag	-	Zinc acetate dehydrate, silver nitrate, distilled water, ammonia solution, tea	300 °C/2 h	Nanoparticles	Dielectric and optical	[57]
Ag	-	Zinc acetate dehydrate, silver nitrate, distilled water, ammonia solution	60 °C/2 h, 300–450 °C/2 h	Nanoparticles	Photoluminescence	[58]
Au	0–1.5 wt%	Zinc acetate dehydrate, gold nitrate, samarium nitrate, deionized water, ammonia solution	80 °C/1 h, 150 °C/12 h, 450 °C/3 h.	Thin film	Photovoltaic, optical	[59]
Au	0.14 wt%	Zinc acetate dehydrate Zn(CH_3_COO)_2_x2H_2_O, HAuCl_4_x3H_2_O, triethanolamine, ethanol	500 °C/3 h	Powder	Optical properties, photocatalytic degradation	[60]
Au	3%	Zn(CH_3_COO)_2_x2H_2_O HAuCl_4_x4H_2_O, ethanol, monoethanolamine (MEA)	650 °C for 6 h	Thin film	Optical properties gas sensing	[61]
Au	10–30 at%	Zn(CH_3_COO)_2_x2H_2_O gold(III) chloride hydrate HAuCl_4_xH_2_O, ethanol, monoethanolamine	50 °C/1 h 200 °C/10 min, 500 °C/1 h.	Thin film	Optical properties, photoluminescence, photocatalytic and sensing properties	[62]
Au	-	Zn(CH_3_COO)_2_x2H_2_O, 2-methoxyethanol, MEA, Gold wires	400 °C/2 h	Thin film	Optical and electrical properties	[63]

## Data Availability

Not applicable.
